# Supplementary material for: PIE-seq: identifying RNA-binding protein targets by dual RNA-deaminase editing and sequencing
Source: Nat Commun. 2023 Jun 6;14:3275. doi: 10.1038/s41467-023-39054-8 (PMC10244410; doi:10.1038/s41467-023-39054-8)
Supplement: Supplementary file 1 — Supplementary Information [file 41467_2023_39054_MOESM1_ESM.pdf]

Supplementary Information for

PIE-Seq: Identifying RNA-binding Protein Targets by Dual RNA-deaminase Editing and Sequencing

Xiangbin Ruan[1], Kaining Hu[1], Xiaochang Zhang[1,*]

[1]Department of Human Genetics and The Neuroscience Institute, University of Chicago, Chicago, USA.

[*]Correspondence: xczhang@uchicago.edu (X.Z.)

The PDF file includes:
Supplementary Figure 1 to 8
Supplementary Note 1 and 2
Supplementary Reference

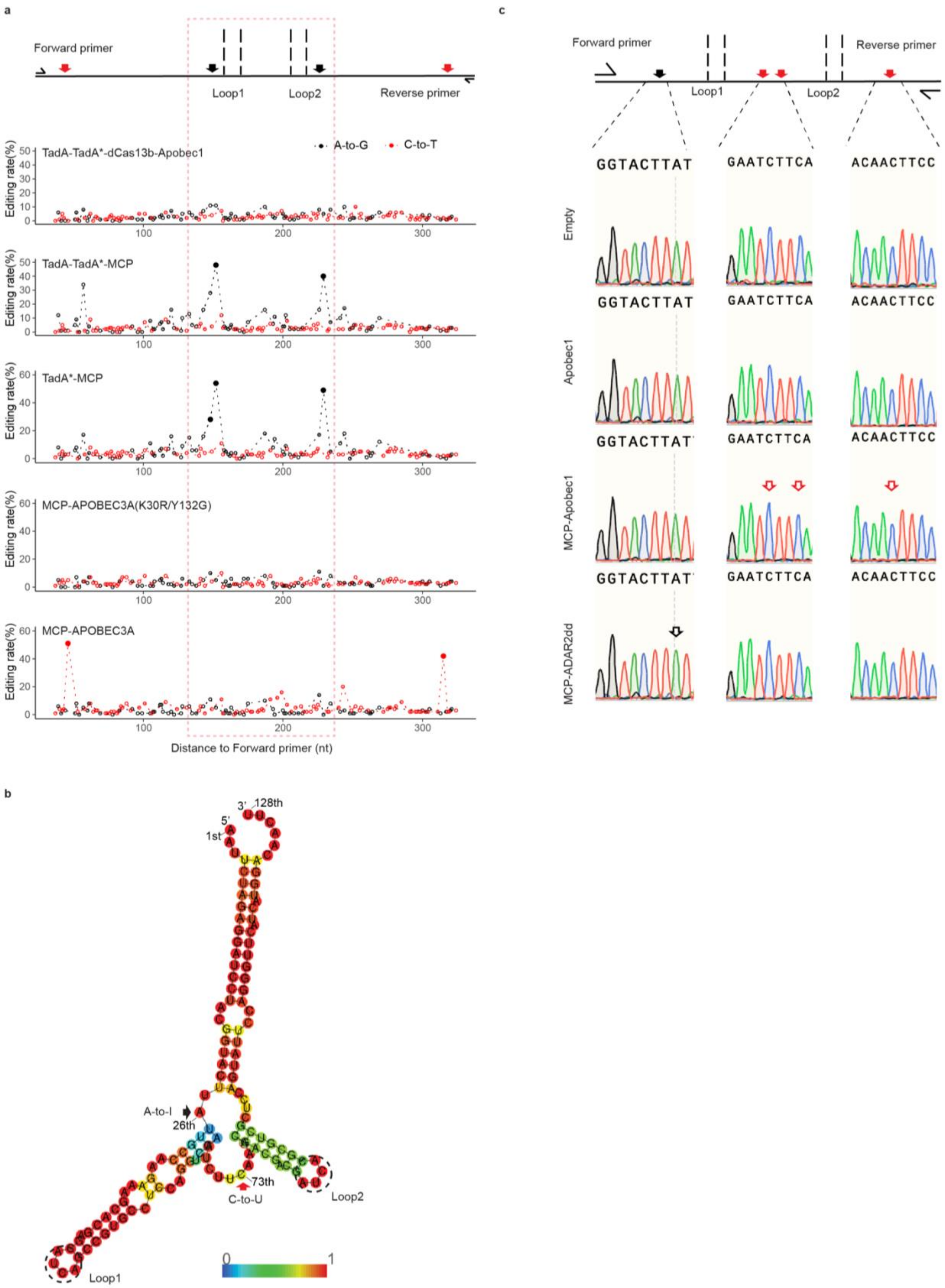

Supplementary Figure 1:The PIE-Seq design and evaluation of deaminase modules.

a Quantification of Sanger sequencing results showing that MCP-Apobec1 and MCP-ADAR2dd introduced A-to-G(I) and C-to-T(U) RNA mutations surrounding MS2 sites (red arrow, C-to-T; black arrow, A-to-G).

b Predicted RNA secondary structure of the 2x MS2 RNA sequences indicating base-pairing probability. The positions with the highest editing rate introduced by MCP-Apobec1 and MCP-ADAR2dd (Fig. 1b) are highlighted by red and black arrows.
c Sanger sequencing results of 2x MS2 sites with DNA extracted from transfected cells showing no observable DNA mutations.

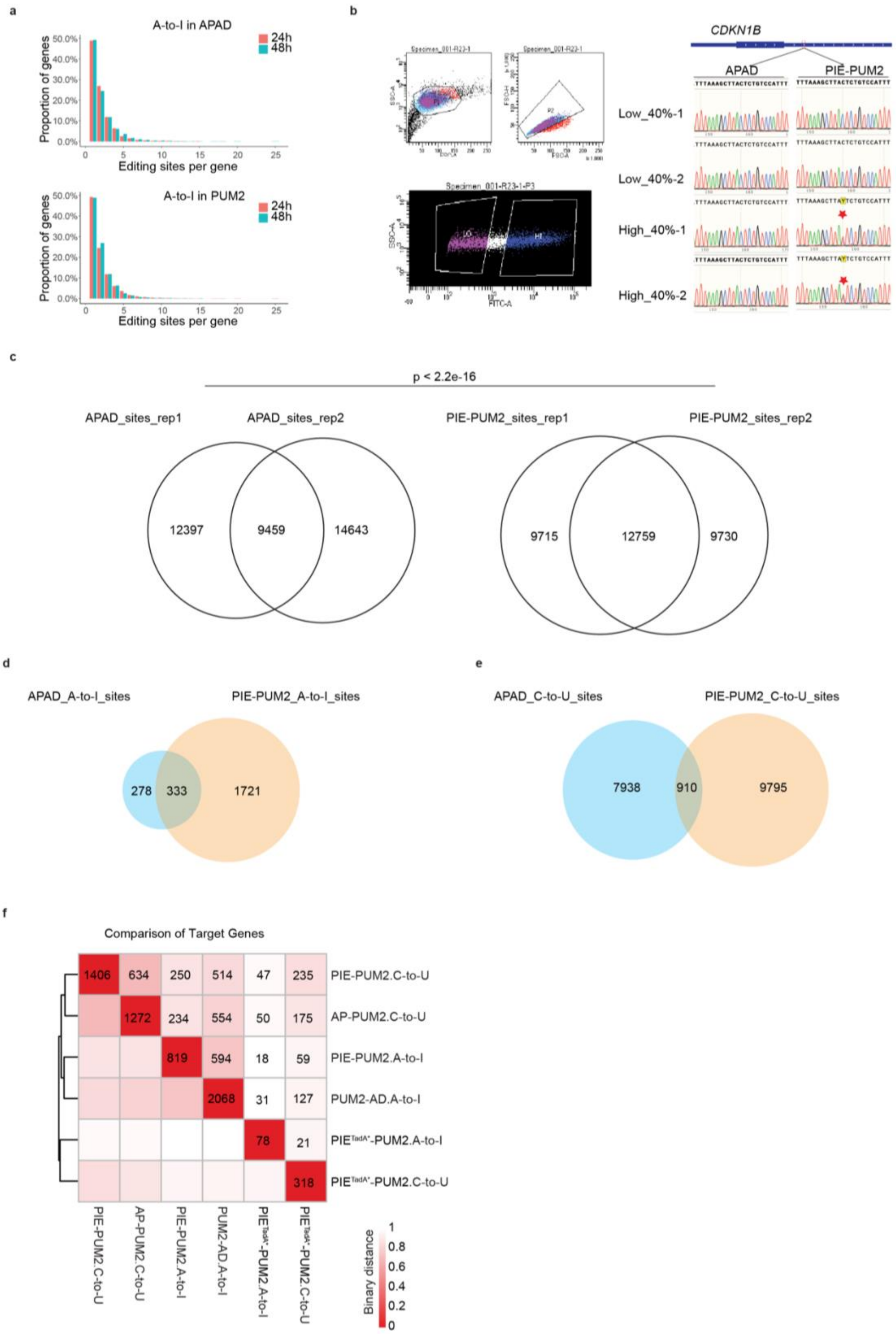

Supplementary Figure 2: Deaminase modules are active in PIE-Seq fusion proteins.

a Bar plots indicating the proportion of genes with indicated numbers of A-to-I editing sites in APAD and PUM2 24h- or 48h-transfection groups.

b Left panel: FACS sorting of high (top 40% of all EGFP positive cells) and low (bottom 40% of all EGFP positive cells) expression of APAD or PIE-PUM2 cells. Right panel: Sanger sequencing results of *CDKN1B* showing that C-to-U editing occurs in EGFP high PIE-PUM2 replicates.

c Venn diagrams showing shared editing sites within APAD replicates (upper panel) and PIE-PUM2 replicates (middle panel) in 24-hour transfection groups. The p-value was calculated by chi-squared test.
d-e Venn diagrams showing shared A-to-I (**d**) and C-to-U (**e**) editing sites between APAD and PIE-PUM2.
f Heatmaps showing the Euclidean distance among target genes identified by PIE-PUM2, PIE^{TadA*}-PUM2, AP-PUM2, and PUM2-AD groups. Smaller value (0-1) indicates a closer distance. The numbers indicate how many target genes were shared between samples.

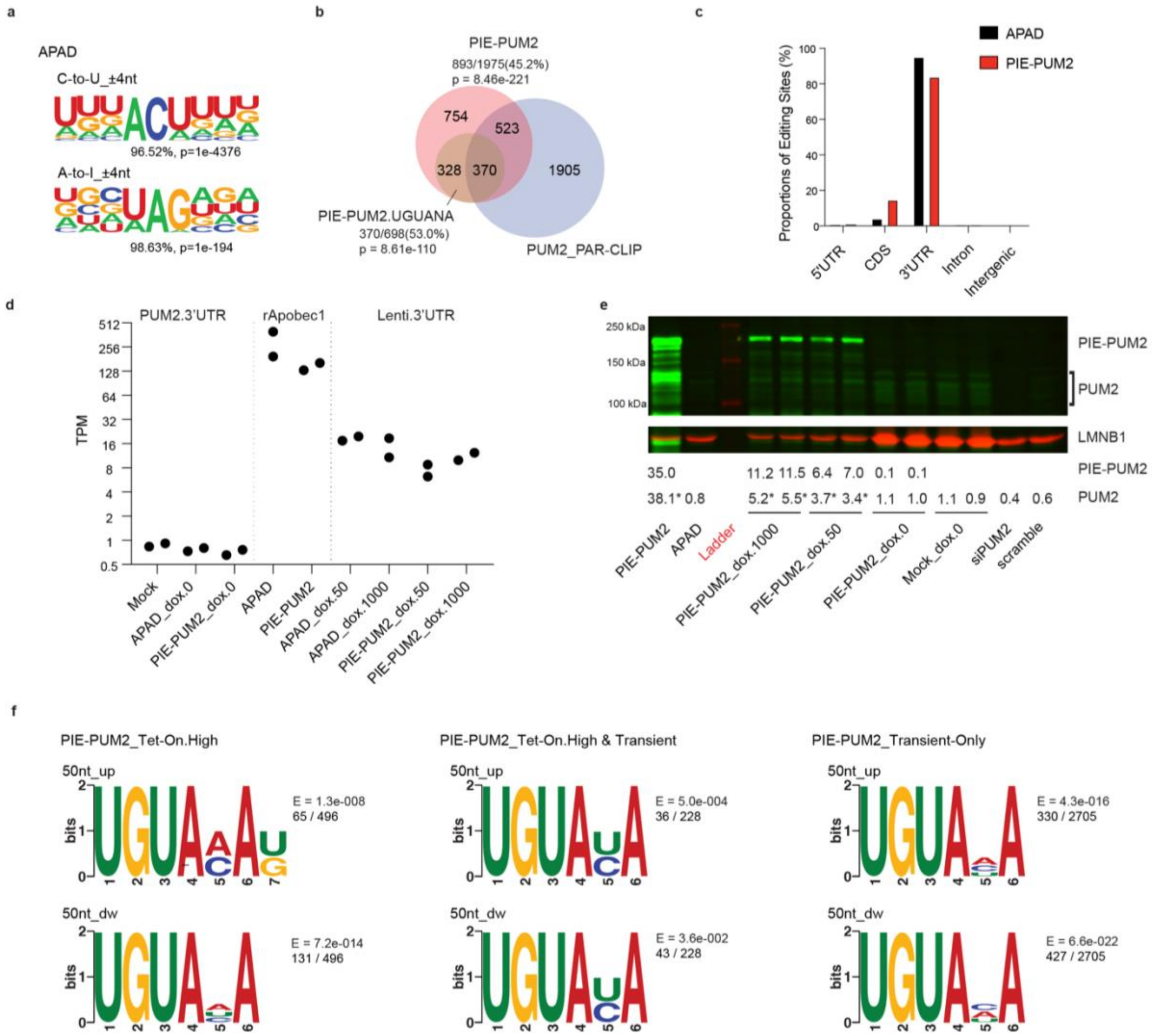

Supplementary Figure 3: PIE-PUM2 identifies PUM2 target genes.

a Nucleobase composition of the ±4-nt region of A-to-I and C-to-U editing sites in APAD.

b Venn diagrams showing comparisons among the target genes identified by PIE-PUM2, targets with target sites surrounding UGUANA motif, and PUM2 PAR-CLIP targets.

c A Bar plot showing the gene region distribution of APAD control sites and PIE-PUM2 target sites. Source data are provided as a Source Data file.

d Differential gene expression analysis showing RNA levels of the endogenous PUM2 (3'UTR) in mock and untreated inducible expression groups, rApobec1 RNA levels in transient expression groups (APAD, PIE-PUM2), and induced PUM2 (Lenti-3'UTR). Each group with two biological replicates. TPM (Transcripts Per Million) values were generated by featureCounts. Source data are provided as a Source Data file.

e Western blot results showing the expression levels of PIE-PUM2 fusion protein and the endogenous PUM2, with LMNB1 as the loading control. *The PIE-PUM2 protein band might affect the endogenous PUM2 signal. The representative image is one from two independently staining experiments. Raw image is provided as a Source Data file.

f The most enriched motifs within the ±50-nt region of Tet-On.High PIE-PUM2 target sites (Left), the shared ones between Tet-On.High and transient PIE-PUM2 (Middle), and the unique target sites in transient PIE-PUM2 group (Right). The E-value is the number of candidate motifs times the enrichment p-value by Fisher's exact test.

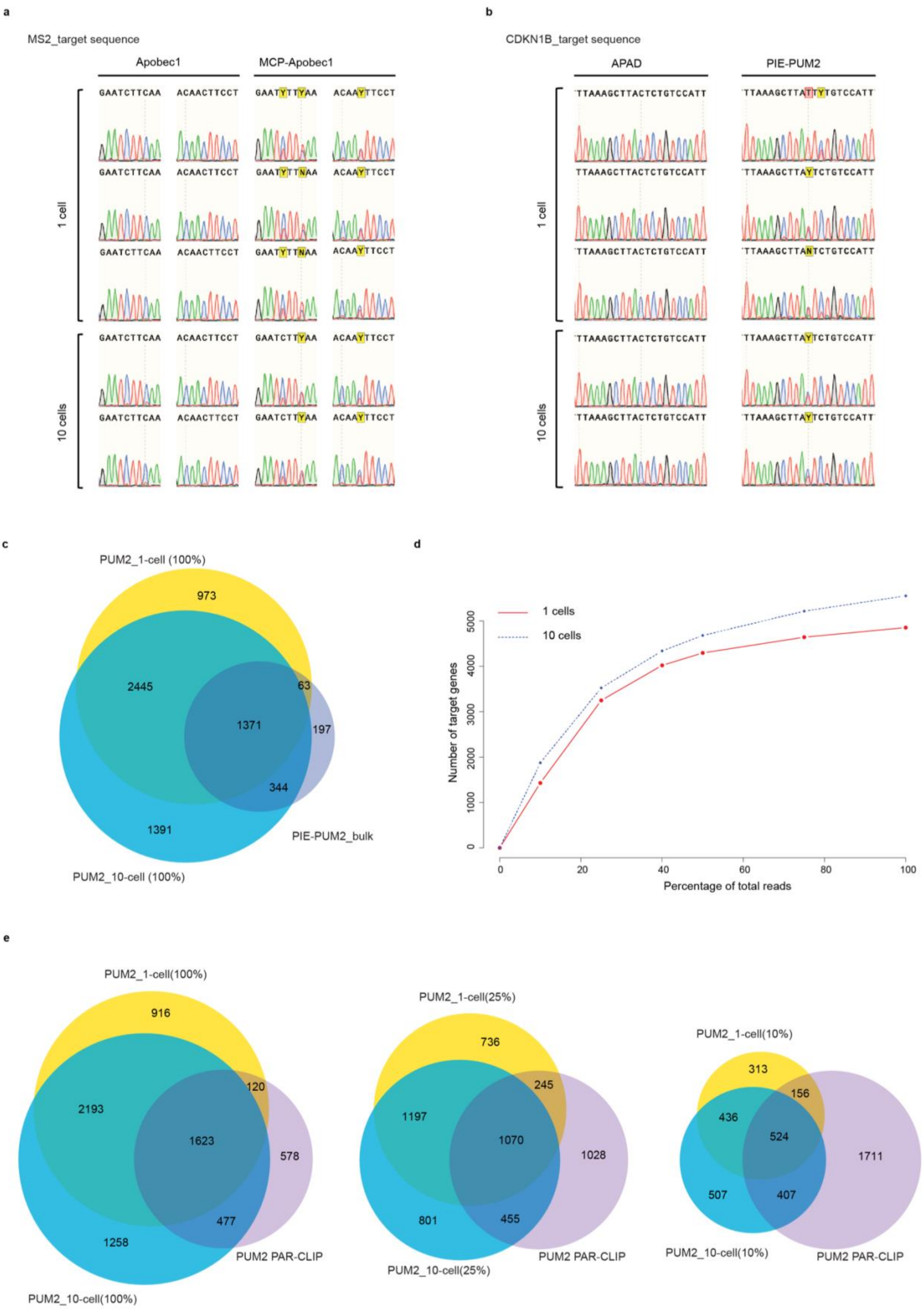

Supplementary Figure 4: Validation of single-cell and 10-cell PIE-PUM2 target sites by Sanger sequencing.

a Sanger sequencing results showing that MCP-Apobec1 introduced C-to-T(U) RNA mutations surrounding MS2 sites in 10-cell and single-cell samples.
b Validation of C-to-U editing site of *CDKN1B* in PIE-PUM2 but not APAD in 10-cell and single-cell samples.
c Venn diagram showing PUM2 targets identified in single-cell and ten-cell samples (80~110 million sequencing reads per sample) largely overlapped with those of bulk PIE-PUM2.
d Line plots showing the number of target genes identified through down-sampling the single- and 10-cell RNA-seq reads by 10%, 25%, 40%, 50%, and 75%. Source data are provided as a Source Data file.
e Venn diagrams showing PUM2 targets identified in single-cell and ten-cell samples and their intersection with those identified by PUM2 PAR-CLIP. 100%, 25%, and 10% indicate the proportions of down-sampling (**d**).

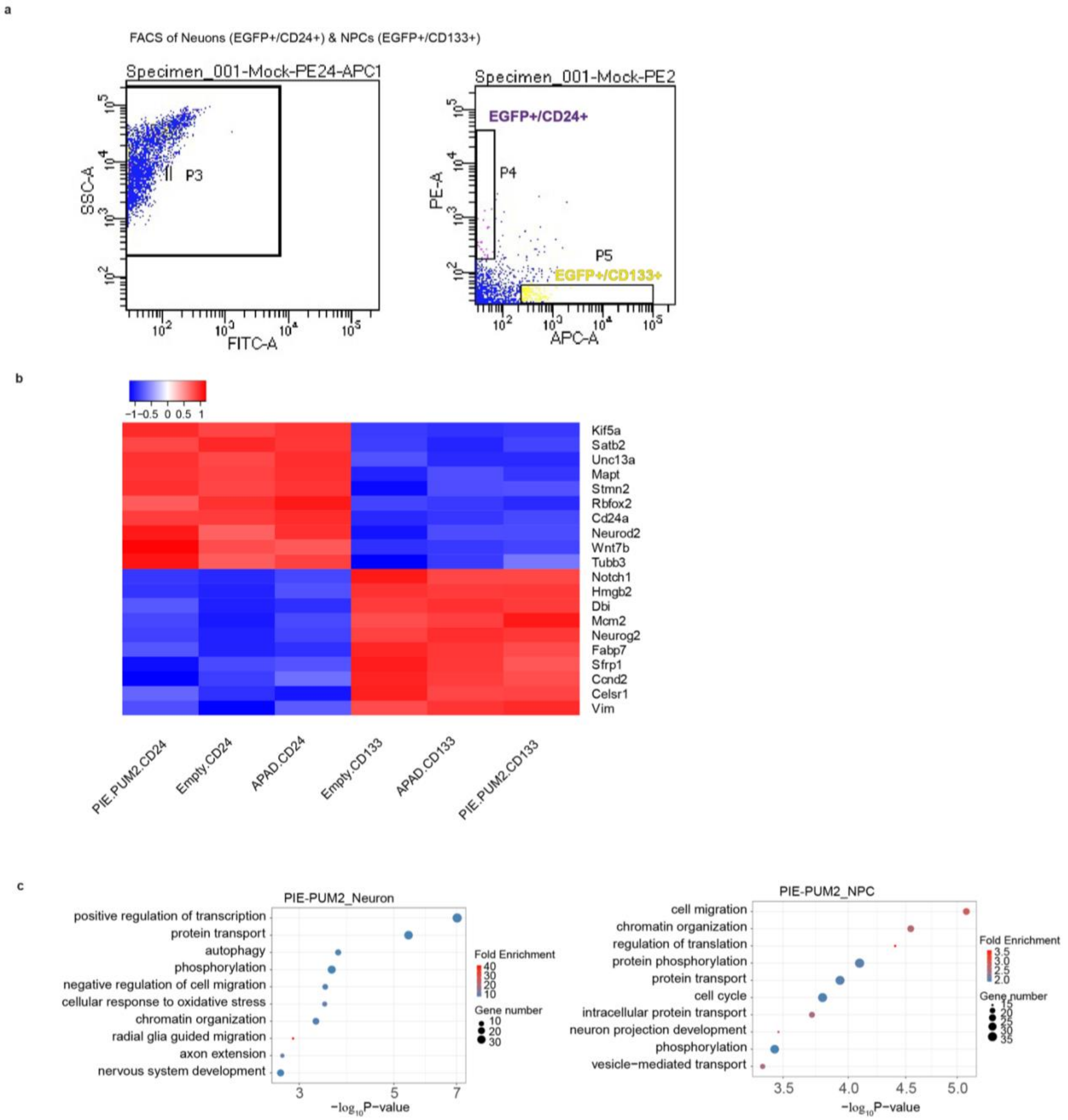

Supplementary Figure 5: PIE-PUM2 identifies mRNA targets in the developing mouse brain.

a FACS sorting for CD24+/EGFP+ or CD133+/EGFP+ dual positive cells from electroporated E15.5 mouse cortical tissues.

b Heatmap for differential gene expression between sorted CD24+ and CD133+ cells in E15.5 empty control, APAD and PIE-PUM2 groups, showing that CD24+ cells were enriched for neuronal genes and that CD133+ cells expressed neural progenitor identity genes. Scale bar shows fold changes of gene expression level (log2 CPM).

c GO analysis of PUM2 target genes identified in PIE-PUM2_Neuron and PIE-PUM2_NPC groups.

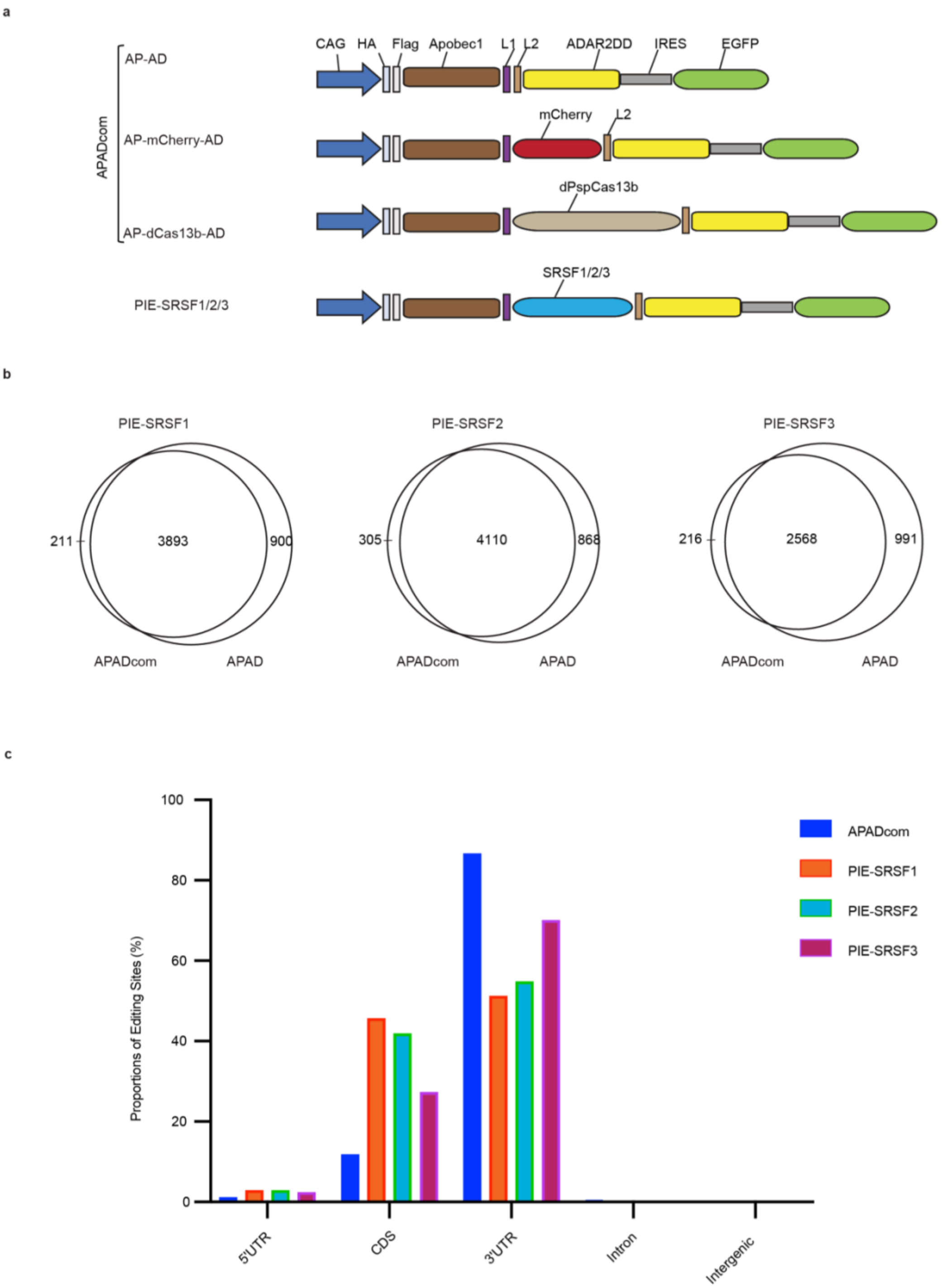

Supplementary Figure 6: Identification of SRSF1/2/3 targets by PIE-Seq.
a Schematics of deaminase controls and the PIE-SRSF1, PIE-SRSF2 and PIE-SRSF3 constructs.
b Venn diagrams comparing PIE-SRSF1/2/3 target genes using APADcom or APAD as the control.
c Bar plot showing gene region distributions of control sites for APADcom and target sites for PIE-SRSF1/2/3 groups. Source data are provided as a Source Data file.

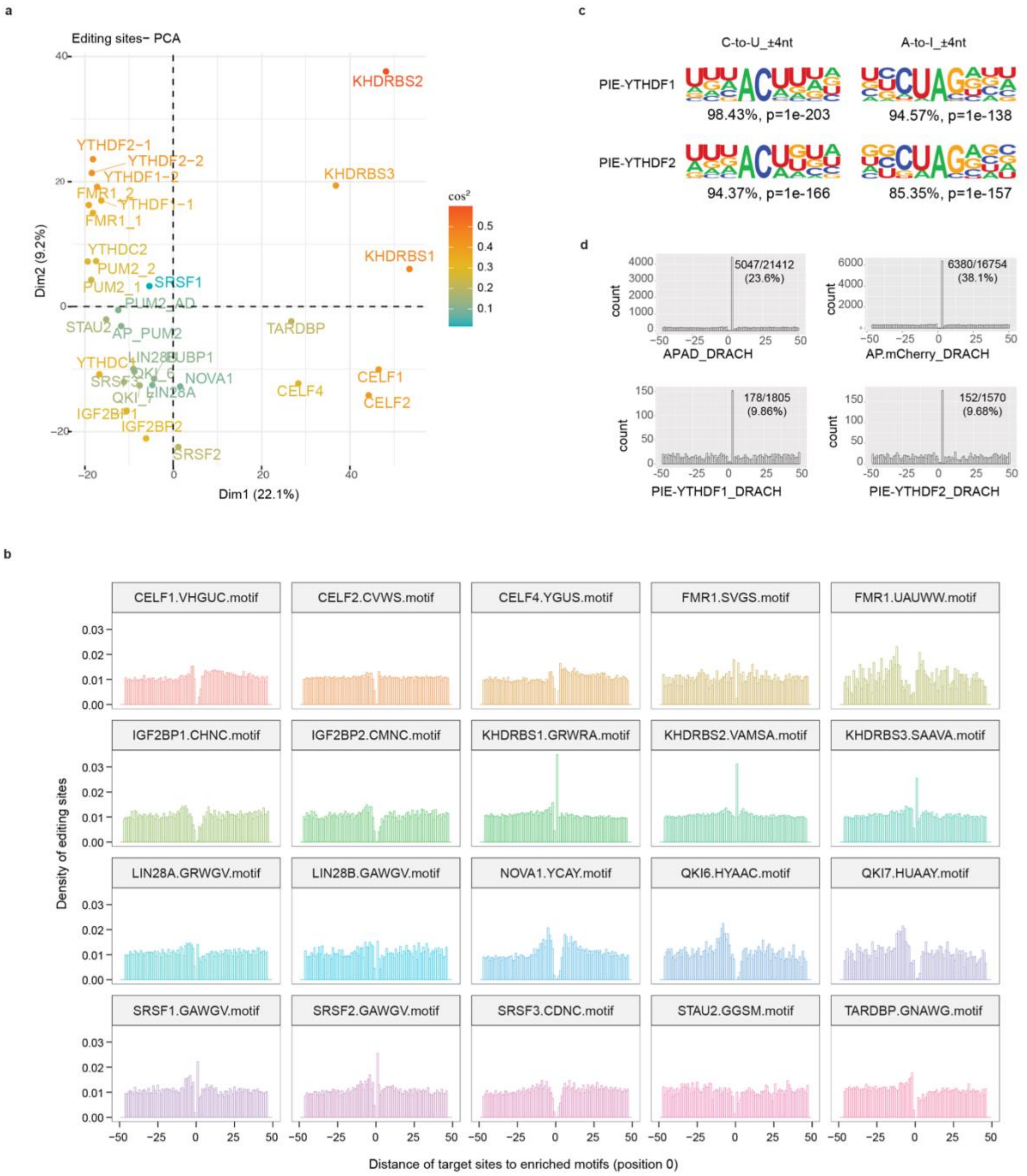

Supplementary Figure 7: PIE-Seq reveals binding features and motifs for human RBPs.

a Principal component analysis (PCA) of raw editing sites (z-score >1, read coverage >=5) for 25 PIE-RBPs.
b The distribution pattern of target sites in ±50nt distance to the presentive binding motifs of each RBP. The length of all motifs was scaled to one and designated position 0.
c Nucleobase composition of the ±4-nt region of A-to-I and C-to-U editing sites in APAD, PIE-YTHDF1 and PIE-YTHDF2, showing that ACU was predominant for C-to-U and UAG was predominant for A-to-I sites.
d Histograms showing that the editing sites were highly enriched in the DRACH motif for all indicated Apobec1 controls and PIE-YTH groups. The ratio indicated the number of editing sites as the "C" in the DRACH motif over the total number of significant edited sites.

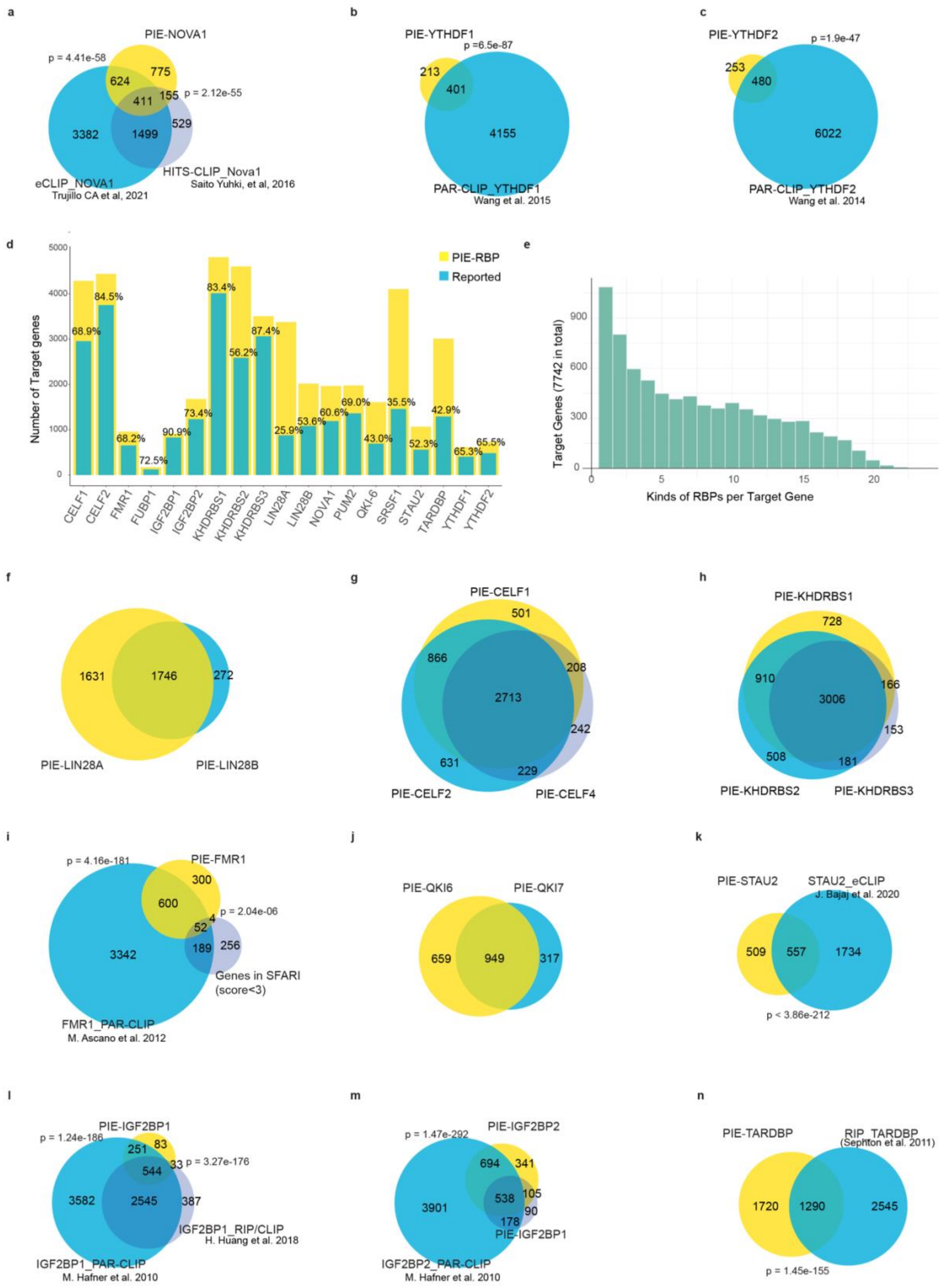

Supplementary Figure 8: PIE-Seq identified high-confidence target genes for human RBPs.
a Venn diagrams showing that NOVA1 target genes identified by PIE-Seq significantly agreed with NOVA1 eCLIP results in human cortical organoids and mNova1 HITS-CLIP on E18.5 mice brain[1,2]. The p-values were calculated with hypergeometric test, and the same method was used for other comparisons in this figure.

b-c Venn diagrams showing target genes identified by PIE-YTHDF1 and PIE-YTHDF2 significantly agree with the YTHDF1and YTHDF2 PAR-CLIP target genes, respectively[3,4].
d A bar plot showing target genes identified by PIE-Seq were cross-validated by previous reports, and nominate new target genes for most tested RBPs. Source data are provided as a Source Data file.
e A bar plot showing the number of share or unique target genes across different RBPs. Source data are provided as a Source Data file.
f Venn diagrams showing that PIE-LIN28A covered most of the PIE-LIN28B targets.
g Venn diagrams showing that CELF1/2/4 PIE-Seq targets genes significantly shared between each other.
h Venn diagrams showing that KHDRBS1/2/3 targets genes identified by PIE-Seq are largely shared.
i Venn diagrams showing that FMR1 targets identified by PIE-Seq significantly overlap with FMR1 CLIP targets and genes implicated in ASD.
j Venn diagrams showing that PIE-QKI7 target genes were largely shared with PIE-QKI6.
k Venn diagrams showing that STAU2 target genes identified by PIE-Seq were significantly validated by STAU2 eCLIP genes in K562 bcCML cells[5].
l-m Venn diagrams showing that IGF2BP1 and IGF2BP2 targets identified by PIE-Seq significantly agree with CLIP and RIP-seq targets. PIE-IGF2BP2 targets included most of the PIE-IGF2BP1 targets.
n Venn diagrams showing that TARDBP target genes identified by PIE-Seq were cross validated with TARDBP/TDP43 RIP-seq targets in rat cortical neurons[6].

Supplementary Note 1: PIE-Seq application on more RBPs

PIE-Seq uncovers target transcripts of QKI-6/7 splice isoforms

RNA-binding proteins are frequently regulated by alternative splicing and generate highly similar protein isoforms, targets of which could be challenging to separate with CLIP-based approaches. QKI has at least three major splice isoforms that differ in their C-termini: only QKI-5 owns a nuclear localization signal (NLS) and functions specifically in the nucleus as a splicing regulator; QKI-6 is distributed throughout the cell and QKI-7 is mainly in the cytosol[7]. PIE-QKI6 and PIE-QKI7 identified 1608 and 1266 target genes, respectively, between which 949 genes are shared (Supplementary Fig. S8j), suggesting that the two QKI isoforms largely bind to the same cohort of target genes. The most enriched motifs identified in the 50-nt upstream and downstream sequences of target sites in PIE-QKI6 and PIE-QKI7 are UAAC, HYAAC, or HUAAY (Supplementary Fig. 7c), which partially overlap with previous reported bipartite QKI response element NACUAAY-N_{1-20}-UAAY[8]. Importantly, a proportion of the QKI6/7 editing sites were immediately upstream of the enriched motifs (Fig. 7d and Supplementary Fig. 7b). These results indicate that PIE-Seq detected target genes and binding motifs of QKI-6/7.

PIE-STAU2 and PIE-IGF2BP1/2 and mRNA transport

RBPs such as STAU2 and IGF2BP1/2 play important roles in mRNA transport, and we seek to determine whether PIE-Seq is applicable to uncover their target genes. STAU2 binds to double-stranded RNA and differentially distributes RNA cargos during asymmetric neural stem cell division[9-11]. We uncovered 1066 STAU2 target genes among which 509 genes were missed by eCLIP in K562 cells[5] (Supplementary Fig. 8k), suggesting the importance of target discovery across different cell types and using orthogonal approaches. The most enriched motifs in flanking sequences of PIE-STAU2 target sites (±50-nt) are GC-rich (Fig. 7c).

IGF2BP1 and IGF2BP2 harbor two RRM and four KH RNA-binding domains, show 65% identity protein sequences with each other, and play direct roles in mRNA transport and translation[12]. PIE-IGF2BP1 identified 911 target genes, with 828 genes cross-validated by previous PAR-CLIP and RIP-seq results (Supplementary Fig. 8l)[13,14]. In parallel, PIE-IGF2BP2 identified 1678 target genes, with 446 genes not identified by PAR-CLIP in HEK293T cells (Supplementary Fig. S8m)[13]. Noticeably, 70.6% of PIE-IGF2BP1 target genes were identified by PIE-IGF2BP2 (Supplementary Fig. S8m). The top enriched motif identified in the flanking sequences of target sites (± 50-nt) were CNNC in both PIE-IGF2BP1 and PIE-IGF2BP2 (Fig. 7c). There are slightly enriched target sites within 10-nt upstream of the CNNC motif both for IGF2BP1 and IGF2BP2 (Fig. 7d and Supplementary Fig. 7b). These results indicate that STAU2 and IGF2BP1/2 bind distinct mRNA motifs.

PIE-Seq uncovers RNA binding motif for FUBP1

Far upstream element binding protein 1 (FUBP1) is initially reported to bind A/T-rich single-stranded DNA (ssDNA) sequence in the c-myc promoter[15], and then reported to also bind AU-rich elements in introns or 3′ UTR of pre-mRNAs[16,17]. Interestingly, the most enriched motif identified by PIE-FUBP1 was UWGUU (Fig. 7c). This new motif is comparable to the TG-rich ssDNA motif previously identified by SELEX for FUBP1[18] and the UGU RNA motif identified for FUBP3 by eCLIP[19], while slightly different from the U-rich motif identified by RBNS assay *in vitro*[20] or FUBP1 easyCLIP[21]. Thus, our PIE-Seq results support that FUBP1 bins to UWGUU motifs.

PIE-Seq uncovers new target genes for TARDBP

TARDBP/TDP-43 preferably binds to tandem UG repeats and plays prominent roles in pathogenesis of neurodegenerative diseases such as ALS[22]. The most enriched motifs for PIE-TARDBP were GAWS and GNAWG (Fig. 7c), resembling loosely conserved UG/GU-rich repeats

interspersed by adenines[23]. *PIE-TARDBP* target sites were enriched upstream (-10nt~-2nt) of the GNAWG motif, and the overall density of upstream target sites is higher than the downstream (Fig. 7d and Supplementary Fig. 7b). The target genes identified by PIE-TARDBP correlated substantially with homologous genes identified by RIP-seq in rat cortical neurons[6], while revealing 1720 human cell-specific target genes (Supplementary Fig. 8n). These results suggest that TARDBP regulates different sets of targets between rodent neurons and human cells.

Supplementary Note 2: PIE-Seq protocol

Reagents and buffers

- pR008_APAD: pCAGIG-HA-Flag-rApobec1-XTEN-hADAR2dd-IRES-EGFP
- pR023_PIE-PUM2: pCAGIG-HA-Flag-rApobec1-XTEN-PUM2-GS-hADAR2dd-IRES-EGFP
- 293FT cell line (Invitrogen, cat. no. R70007) The cell line has been checked that it is not infected with mycoplasma.
- DMEM culture media (Gibco cat. No. 10566024)
- Fetal bovine serum (Gibco, cat. No. 26140079)
- DPBS without Calcium and Magnesium (Thermo Fisher Scientific, cat. No. 14-190-250)
- Trypsin-EDTA (0.25%), phenol red (Fisher Scientific, cat. No. 25200114)
- Opti MEM I Reduced Serum Medium (Fisher Scientific, cat. No. 31-985-062)
- Lipofectamine 2000 (Fisher Scientific cat. No. 11668019)
- TRIzol Reagent (Thermo Fisher Scientific, cat. No. 15596018)
- Direct-zol RNA MicroPrep Kit (Zymo Research, cat. No. R2060)
- Ethanol, 200 proof (100%) (Thermo Fisher Scientific, cat. No. 04355451)
- TruSeq RNA Library Preparation Kit v2 (Illumina, cat. No. RS1222001)
- Agencourt AMPure XP beads (Beckman Coulter, cat. No. A63880)
- Invitrogen SuperScript IV Reverse Transcriptase (Thermo Fisher Scientific, cat. No. 18090050)
- Agilent RNA 6000 Nano Kit (Agilent, cat. No. 5067-1511)
- Agilent High Sensitivity DNA Kit (Agilent, cat. No. 5067-4626)
- TruSeq Stranded mRNA Library Prep (48 Samples) (Illumina, cat. No. 20020594)

Equipment

- 100mm Tissue Culture Dish (TPP, cat. No. 93100)
- 12 Well TC MULTIDISH (Fisher Scientific cat. No. FB012928)
- Cell Strainer 70UM (Fisher Scientific, cat. No. 22363548)
- Microcentrifuge Tubes: 1.5mL (Thermo Fisher Scientific, cat. No. 05408129)
- Falcon 15mL Conical Centrifuge Tubes (Fisher Scientific, cat. No. 352097)
- UltraFlux SnapStrip PCR tube, 8-strip, 0.2mL (SSIbio, cat. No. 3240-00S)
- 96M super magnet plate（E & K Scientific, cat. No. EK-29015）
- Flow cytometer (BD Biosciences, model No. FACSAria IIu)
- Spectrophotometer (Thermo Fisher Scientific, model No. NanoDrop ND-1000)
- Electrophoresis instrument (Agilent, model No. 2100 Bioanalyzer)
- Sequencing system (Illumina, model No. NextSeq 500)

Software

- Linux environment
- Java(v1.8.0): https://jdk.java.net/archive/
- Python (v2.7.15): https://www.python.org/downloads/
- R(v 4.2.0)/RStudio (v 1.1.456): https://www.rstudio.com
- Perl (v5.24.1): https://www.perl.org/get.html
- fastp: https://github.com/OpenGene/fastp
- STAR (v2.6.1b): https://github.com/alexdobin/STAR
- Picard: https://broadinstitute.github.io/picard/
- samtools (v1.9): http://samtools.sourceforge.net/
- bedtools (v2.27.1): http://bedtools.readthedocs.io/
- JACUSA (v2.0.0): https://github.com/dieterich-lab/JACUSA2
- DREME: https://meme-suite.org/meme/doc/dreme.html

- Homer: http://homer.ucsd.edu/homer/motif/
-

PIE-Seq workflow

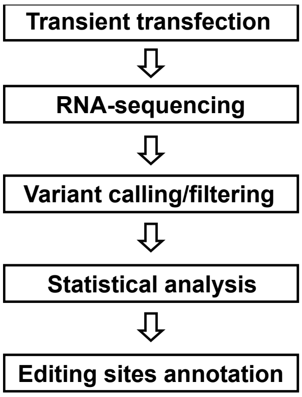

Transient transfection of 293FT cells with PIE-RBP and control plasmids

Notes before starting:

1) Growth the 293FT cells (70-80%)

2) All steps can be done at room temperature

3) Medium should be prewarmed or at least at RT

4) Prepare 500ul of prewarmed medium in each well of a 12-well plate

1. Dilute 1.0 µg of plasmids DNA in Opti-MEM I medium to a total volume of 20ul as follows.

   Note: To keep low expression level of PIE-RBP and increase transfection efficiency, we co-transfect low dose of PIE-Seq plasmids with helper vector (pCRII-TOPO).

   - Mock control group: pCAGIG (200ng) + pCRII-TOPO (800ng)
   - Non-targeting group: pR008 (260ng) + pCRII-TOPO (740ng)
   - PIE-PUM2 group: pR023 (360ng) + pCRII-TOPO (640ng)

2. Dilute 2.0 µl of lipofectamine 2000 reagent in Opti-MEM I medium to a total volume of 40ul. Mix gently and incubate at room temperature for 5 minutes.
3. Combine Lipofectamine dilution and DNA dilution together
4. Incubate the mixtures for 15 minutes at room temperature.
5. In the meantime, prepare 293FT cells:

   1) Wash the cells with DPBS

   2) Add 2ml Trypsin to a 100mm plate and incubate at 37°C for 2min

   3) Stop with 2ml of DMEM/FBS medium (no pen/strep), pipette the suspension to a 15ml tube and mix by pipetting up and down

   4) Centrifuge cells at 1000rpm for 3min

   5) Resuspend cells in 5 mL media (no pen/strep)

6) Dilute 4.0x10^6 cells in 1ml, so that you finally have 8.0x10^5 cells in 200µl medium.

6. Add 200 µl cells to transfection mixture.
7. Add mixture of cells and transfection solution into each well in 12-well plate. Culture at 37°C incubator.
8. Change medium 4 hours after transfection.
9. Harvest cells after 24 or 48 hours.

FACS sorting of 293FT cells (for transfecting 24-hour cells)

1. Wash the cells with DPBS once.
2. Add 2ml Trypsin to a 100mm plate and incubate at 37°C for 2min.
3. Stop with 2ml of DMEM/FBS medium, pipette the suspension to a 15ml tube and gently mix by pipetting up and down.
4. Centrifuge cells at 1000rpm for 3min.
5. Resuspend cells with 1ml DPBS (containing 1% BSA).
6. Filter cells with 70-µm cell strainer.
7. Incubate cells on ice before sorting.
8. Sorting top 40% EGFP positive cells into 500 µl TRIzol regent.

Extract total RNA and reverse transcript PCR

Extract total RNA from transfection cells with Direct-zol RNA MicroPrep Kit (Zymo Research, cat. No. R2060) following its standard protocol.

Reverse transcript PCR system is optimized as follows.

1. Combine the following components in a reaction tube

Component	Volume
50 µM random hexamers	0.5 µL
10 mM dNTP mix (10 mM each)	0.5 µL
Total RNA	300ng-1 µg total RNA
DEPC-treated water	to 6.5 µL

2. Mix and briefly centrifuge the components.

3. Heat the RNA-primer mix at 65°C for 5 minutes, and then incubate on ice for at least 1 minute.

4. Combine the following components in a reaction tube.

Component	Volume
5X SSIV Buffer	2 µL
100 mM DTT	0.5 µL
RNaseOUT Recombinant RNase Inhibitor 1 µL	0.5 µL
SuperScript. IV Reverse Transcriptase (200 U/µL) 1 µL	0.5 µL

5.Cap the tube, mix, and then briefly centrifuge the contents.

6.Add RT reaction mix to the annealed RNA.
7. 23 °C for 10 min, 50 °C for 15 min, 80 °C for 10 min.
8. Dilute cDNA with distilled water at the ratio of 1:10 or 1:20 for subsequent PCR and Sanger sequencing.

Preparation of RNA-Seq libraries

1. Prepare bulk RNA-Seq libraries with TruSeq Stranded mRNA Library Prep (Illumina, cat. No. 20020594)

   We followed step-by-step illumina protocol (https://support.illumina.com/content/dam/illumina-support/documents/documentation/chemistry_documentation/samplepreps_truseq/truseq-stranded-mrna-workflow/truseq-stranded-mrna-workflow-reference-1000000040498-00.pdf).

   For the protocol is optimized for 0.1–1 μg of total RNA, we used 100-300 ng of total RNA as input.

Editing site and target gene analysis

Variants Calling

1. Preprocess paired Fastq files with fastp tool

   ```
   fastp -i Sample_R1.fastq.gz -I Sample_R2.fastq.gz -o Sample_R1.trimmed.fastq.gz -O Sample_R2.trimmed.fastq.gz --detect_adapter_for_pe -x -q 25 -n 1 -l 50 -y -w 16
   ```
2. Align and map Fastq files to hg38 with STAR, and output bam files.

   ```
   STAR --twopassMode Basic --runThreadN 16 --readFilesCommand zcat --genomeDir hg38_STAR_index_UCSC  --genomeLoad NoSharedMemory --outFileNamePrefix Sample_ --outReadsUnmapped Fastx --outSAMtype BAM SortedByCoordinate --outSAMstrandField intronMotif --outSAMattributes All --outFilterType BySJout --outFilterMultimapNmax 1 --alignSJoverhangMin 8 --alignSJDBoverhangMin 1 --outFilterMismatchNmax 999 --outFilterMismatchNoverLmax 0.04 --alignIntronMin 20 --alignIntronMax 1000000 --alignMatesGapMax 1000000 --readFilesIn Sample_R1.fastq.gz  Sample_R2.fastq.gz
   ```
3. Locate and tag duplicate reads in bam file with MarkDuplicates (Picard).

   ```
   java -Xmx8G -jar picard.jar MarkDuplicates I= Sample_Aligned.sortedByCoord.out.bam O= Sample_dup.bam M= Sample.rmdup.log REMOVE_DUPLICATES=true ASSUME_SORTED=true VALIDATION_STRINGENCY=LENIENT
   ```
4. Calling RNA variants from control and PIE-RBP bam files with JACUSA.

   ```
   java -jar JACUSA_v2.0.0.jar call-2 -s -c 5 -P RF-FIRSTSTRAND -p 180 -W 1000000 -F 1024 -filterNM_1 7 -filterNM_2 7 -T 1 -a D,Y -r Control.vs.PIE-RBP.all.editing.sites Control_rep1_dup.bam,Control_rep2_dup.bam RBP_rep1_dup.bam,RBP_rep2_dup.bam
   ```
5. Filtering out of known snp from dbsnp_138.hg38 (ftp://gsapubftp-anonymous@ftp.broadinstitute.org/bundle/hg38/dbsnp_138.hg38.vcf.gz) from *Control.vs.PIE-RBP.all.editing.sites* table with bedtools intersect to get *Control.vs.PIE-RBP.DP5.wo.snp* table.

Comparing editing sites across groups with JACUSA2helper

1. Filter out endogenous editing sites identified in mock group from PIE-RBP groups in *Control.vs.PIE.RBP.DP5.wo.snp* to get *Control.vs.PIE.RBP.wo.bg* table.
2. Select "C->T" and "A->G" mutation sites across non-targeting and *PIE-RBP group* to get *Control.vs.PIE.RBP.edit*.
3. Add editing rates for each replicate in non-targeting and PIE-RBP group to *Control.vs.PIE.RBP.edit*.
4. Add average editing rate for non-targeting and PIE-RBP group to *Control.vs.PIE.RBP.edit*.
5. Add delta rate to *Control.vs.PIE.RBP.edit* by (PIE-RBP – APAD) average editing rate.
6. Assign z-score directions for each editing site according to differential editing rate (z-score value will be positive if delta editing rate (PIE-PUM2 – APAD) > 0, otherwise negative).
7. Annotate editing sites with hg38.refGene.gtf with gene symbol to get the *Control.vs.PIE.RBP.ann*.
8. Define the editing sites with significantly different mutation frequencies between APAD and PIE-RBP as target sites :log-likelihood z-score >= 4, editing rate >= 5 %, editing rate in PIE-RBP >= 2 folding of in APAD control), and then filter out non-target sites to get *Control.vs.PIE.RBP.master*.
9. Genes with target sites are considered as candidate RBP target genes, the “target” sites in APAD (comparing to Empty group) were defined as control sites as background for motif analysis.

Identification of RNA motifs

1. Plot RNA deaminases editing motifs (± 4-nt of editing sites) with HOMER
   findMotifs.pl -rna -len 9
2. Identify de novo motifs flanking editing sites (±50-nt) with DREME
   dreme -p -o -rna

Supplementary Reference

1 Saito, Y. *et al.* NOVA2-mediated RNA regulation is required for axonal pathfinding during development. *Elife* **5**, e14371 (2016).

2 Trujillo, C. A. *et al.* Reintroduction of the archaic variant of NOVA1 in cortical organoids alters neurodevelopment. *Science* **371**, eaax2537 (2021).

3 Wang, X. *et al.* N6-methyladenosine modulates messenger RNA translation efficiency. *Cell* **161**, 1388-1399 (2015).

4 Wang, X. *et al.* N6-methyladenosine-dependent regulation of messenger RNA stability. *Nature* **505**, 117-120 (2014).

5 Bajaj, J. *et al.* An in vivo genome-wide CRISPR screen identifies the RNA-binding protein Staufen2 as a key regulator of myeloid leukemia. *Nature cancer* **1**, 410-422 (2020).

6 Sephton, C. F. *et al.* Identification of Neuronal RNA Targets of TDP-43-containing Ribonucleoprotein Complexes*♦. *Journal of biological chemistry* **286**, 1204-1215 (2011).

7 Zhao, L., Mandler, M. D., Yi, H. & Feng, Y. Quaking I controls a unique cytoplasmic pathway that regulates alternative splicing of myelin-associated glycoprotein. *Proceedings of the National Academy of Sciences* **107**, 19061-19066 (2010).

8 Galarneau, A. & Richard, S. Target RNA motif and target mRNAs of the Quaking STAR protein. *Nature structural & molecular biology* **12**, 691-698 (2005).

9 Vessey, J. P. *et al.* An asymmetrically localized Staufen2-dependent RNA complex regulates maintenance of mammalian neural stem cells. *Cell stem cell* **11**, 517-528 (2012).

10 Heraud-Farlow, J. E. *et al.* Staufen2 regulates neuronal target RNAs. *Cell reports* **5**, 1511-1518 (2013).

11 Chowdhury, R. *et al.* STAU2 binds a Complex RNA Cargo that changes temporally with production of diverse intermediate progenitor cells during mouse corticogenesis. *Development* **148**, dev199376 (2021).

12 Degrauwe, N., Suvà, M.-L., Janiszewska, M., Riggi, N. & Stamenkovic, I. IMPs: an RNA-binding protein family that provides a link between stem cell maintenance in normal development and cancer. *Genes & development* **30**, 2459-2474 (2016).

13 Hafner, M. *et al.* Transcriptome-wide identification of RNA-binding protein and microRNA target sites by PAR-CLIP. *Cell* **141**, 129-141 (2010). https://doi.org:10.1016/j.cell.2010.03.009

14 Huang, H. *et al.* Recognition of RNA N 6-methyladenosine by IGF2BP proteins enhances mRNA stability and translation. *Nature cell biology* **20**, 285-295 (2018).

15 Braddock, D. T., Louis, J. M., Baber, J. L., Levens, D. & Clore, G. M. Structure and dynamics of KH domains from FBP bound to single-stranded DNA. *Nature* **415**, 1051-1056 (2002).

16 Zhang, J. & Chen, Q. Far upstream element binding protein 1: a commander of transcription, translation and beyond. *Oncogene* **32**, 2907-2916 (2013).

17 Jacob, A. G., Singh, R. K., Mohammad, F., Bebee, T. W. & Chandler, D. S. The splicing factor FUBP1 is required for the efficient splicing of oncogene MDM2 pre-mRNA. *Journal of Biological Chemistry* **289**, 17350-17364 (2014).

18 Benjamin, L. R. *et al.* Hierarchical mechanisms build the DNA-binding specificity of FUSE binding protein. *Proceedings of the National Academy of Sciences* **105**, 18296-18301 (2008).

19 Van Nostrand, E. L. *et al.* A large-scale binding and functional map of human RNA-binding proteins. *Nature* **583**, 711-719 (2020). https://doi.org:10.1038/s41586-020-2077-3

20 Dominguez, D. *et al.* Sequence, structure, and context preferences of human RNA binding proteins. *Mol Cell* **70**, 854-867. e859 (2018).

21 Porter, D. F. *et al.* easyCLIP analysis of RNA-protein interactions incorporating absolute quantification. *Nature communications* **12**, 1-16 (2021).

22 Tollervey, J. R. *et al.* Characterizing the RNA targets and position-dependent splicing regulation by TDP-43. *Nature neuroscience* **14**, 452-458 (2011).

23 Bhardwaj, A., Myers, M. P., Buratti, E. & Baralle, F. E. Characterizing TDP-43 interaction with its RNA targets. *Nucleic acids research* **41**, 5062-5074 (2013).
