# Supplementary material for: PIE-seq: identifying RNA-binding protein targets by dual RNA-deaminase editing and sequencing
Source: Nat Commun. 2023 Jun 6;14:3275. doi: 10.1038/s41467-023-39054-8 (PMC10244410; doi:10.1038/s41467-023-39054-8)
Supplement: Supplementary file 3 — Description of Additional Supplementary Files [file 41467_2023_39054_MOESM3_ESM.pdf]

Description of Additional Supplementary Files

Supplementary Data 1 : Primer sequences for cloning PIE-RBP plasmids and PUM2 RIP-qPCR.

Supplementary Data 2 : Target sites and target genes for 25 RBPs identified by PIE-Seq in HEK293FT cells. Related to Figure 7 and Supplementary Figure 8.

Supplementary Data 3 : Enriched sequence motifs for 25 RBPs identified by PIE-Seq, and the comparisons to previously reported ones. Related to Figure 7.
