# Supplementary material for: PIE-seq: identifying RNA-binding protein targets by dual RNA-deaminase editing and sequencing
Source: Nat Commun. 2023 Jun 6;14:3275. doi: 10.1038/s41467-023-39054-8 (PMC10244410; doi:10.1038/s41467-023-39054-8)
Supplement: Supplementary file 7 — Reporting Summary [file 41467_2023_39054_MOESM7_ESM.pdf]

nature portfolio

Corresponding author(s): Xiaochang Zhang

Last updated by author(s): May 18, 2023

Reporting Summary

Nature Portfolio wishes to improve the reproducibility of the work that we publish. This form provides structure for consistency and transparency in reporting. For further information on Nature Portfolio policies, see our Editorial Policies and the Editorial Policy Checklist.

Statistics

For all statistical analyses, confirm that the following items are present in the figure legend, table legend, main text, or Methods section.

n/a	Confirmed	
☐	☒	The exact sample size (*n*) for each experimental group/condition, given as a discrete number and unit of measurement
☐	☒	A statement on whether measurements were taken from distinct samples or whether the same sample was measured repeatedly
☐	☒	The statistical test(s) used AND whether they are one- or two-sided<br>*Only common tests should be described solely by name; describe more complex techniques in the Methods section.*
☒	☐	A description of all covariates tested
☐	☒	A description of any assumptions or corrections, such as tests of normality and adjustment for multiple comparisons
☐	☒	A full description of the statistical parameters including central tendency (e.g. means) or other basic estimates (e.g. regression coefficient) AND variation (e.g. standard deviation) or associated estimates of uncertainty (e.g. confidence intervals)
☐	☒	For null hypothesis testing, the test statistic (e.g. *F*, *t*, *r*) with confidence intervals, effect sizes, degrees of freedom and *P* value noted<br>*Give P values as exact values whenever suitable.*
☒	☐	For Bayesian analysis, information on the choice of priors and Markov chain Monte Carlo settings
☒	☐	For hierarchical and complex designs, identification of the appropriate level for tests and full reporting of outcomes
☒	☐	Estimates of effect sizes (e.g. Cohen's *d*, Pearson's *r*), indicating how they were calculated

Our web collection on statistics for biologists contains articles on many of the points above.

Software and code

Policy information about availability of computer code

Data collection	Bulk sequencing libraries were prepared with TruSeq Stranded mRNA Library Prep kit (Illumina), except that NOVA1, FUBP1, TARDBP, CELF1/2/4, and KHDRBS1/2/3 were prepared with Stranded Total RNA Prep kit (Illumina). Each sample was barcoded using IDT for Illumina-TruSeq RNA UD Indexes (IDT). Ten- and Single-cell libraries were made following Smart-seq2 protocol. Libraries were sequenced on the Illumina Next-SEQ500 with 75bp pair-end runs or Nova-Seq with 60bp pair-end runs by the University of Chicago Genomics facility.
Data analysis	DGE analysis from RNA-seq data followed the fastp (v0.23.2), STAR (v2.6.1b), and DESeq2 (human samples), or Rsubread (v3.14) and limma (v3.42.1) (mouse samples) workflows.<br>RNA variant calling pipeline with JACUSA2 package (v2.0.0-RC23).<br>Duplicate reads were identified with MarkDuplicates tool in Picard (version:2.19.1).<br>The RBP target sites (C-to-T or A-to-G) were further analyzed with JACUSA2helper-1.99-9200 in R 4.2.0.<br>The deaminases editing sequence motifs (±4-nt flanking editing sites) were generated with HOMER (v4.11).<br>Genomic distribution of target sites was analyzed with the CTK package.<br>Enriched motifs for PIE-RBPs were identified by DREME(v5.4.1).<br>Phylogenetic tree for RBP proteins was plot with UPGMA (unweighted pair group method with arithmetic mean) method in MEGA11.

For manuscripts utilizing custom algorithms or software that are central to the research but not yet described in published literature, software must be made available to editors and reviewers. We strongly encourage code deposition in a community repository (e.g. GitHub). See the Nature Portfolio guidelines for submitting code & software for further information.

Data

Policy information about availability of data

All manuscripts must include a data availability statement. This statement should provide the following information, where applicable:

- Accession codes, unique identifiers, or web links for publicly available datasets
- A description of any restrictions on data availability
- For clinical datasets or third party data, please ensure that the statement adheres to our policy

All raw and processed sequencing data generated in this study have been submitted to the NCBI Gene Expression Omnibus (GEO; https://www.ncbi.nlm.nih.gov/geo/) under accession number GSE155844: https://www.ncbi.nlm.nih.gov/geo/query/acc.cgi?acc=GSE155844. There is no clinical datasets, or third party data, or restriction on data availability. RNA-seq data for QKI-6, IGF2BP1, IGF2BP2 PAR-CLIP from GEO/GSE21578 (https://www.ncbi.nlm.nih.gov/geo/query/acc.cgi?acc=GSE21578), RNA-seq data for FMR1 PAR-CLIP from GEO/GSE39686 (https://www.ncbi.nlm.nih.gov/geo/query/acc.cgi?acc=GSE39686), NOVA1 eCLIP RNA-seq data from BioProject under accession code PRJNA670687 (https://www.ncbi.nlm.nih.gov/bioproject/?term=PRJNA670687) and mouse Nova1 HITS-CLIP RNA-seq data from GSE69711 (https://www.ncbi.nlm.nih.gov/geo/query/acc.cgi?acc=GSE69711).

Human research participants

Policy information about studies involving human research participants and Sex and Gender in Research.

Reporting on sex and gender	N/A
Population characteristics	N/A
Recruitment	N/A
Ethics oversight	N/A

Note that full information on the approval of the study protocol must also be provided in the manuscript.

Field-specific reporting

Please select the one below that is the best fit for your research. If you are not sure, read the appropriate sections before making your selection.

☒ Life sciences ☐ Behavioural & social sciences ☐ Ecological, evolutionary & environmental sciences

For a reference copy of the document with all sections, see nature.com/documents/nr-reporting-summary-flat.pdf

Life sciences study design

All studies must disclose on these points even when the disclosure is negative.

Sample size	The average library size for each sample or replicate is 48.5 million 75bp or 60bp paired-end reads, which is enough for RNA variants calling according to our down-sampling analysis results and the previous variants calling report.
Data exclusions	No data were excluded from the analysis.
Replication	Two replicates for mock control, APAD control, PIE-PUM2, Dox-inducing APAD and PIE-PUM2, 10-cell APAD and PIE-PUM2, PIE-FMR1, PIE-YTHDF1, PIE-YTHDF2 have two replicates; Three replicates for single-cell APAD, single-cell PIE-PUM2; Other samples were from single experiment. Our analysis support that PUM2 single sample RNA-seq data provide sufficient confidence in calling target genes. These additional samples were derived from different transfection/library batches but were pooled together in the same sequencing experiment. As a result, they can serve as internal controls for each other in our PCA analysis.
Randomization	Total RNA of each sample were collected and sequenced together without randomization.
Blinding	It is clear that RNA data comes from which specific RBP or control group - there was no blinding in this study.

Reporting for specific materials, systems and methods

We require information from authors about some types of materials, experimental systems and methods used in many studies. Here, indicate whether each material, system or method listed is relevant to your study. If you are not sure if a list item applies to your research, read the appropriate section before selecting a response.

Materials & experimental systems

n/a	Involved in the study
☐	☒ Antibodies
☐	☒ Eukaryotic cell lines
☒	☐ Palaeontology and archaeology
☐	☒ Animals and other organisms
☒	☐ Clinical data
☒	☐ Dual use research of concern

Methods

n/a	Involved in the study
☒	☐ ChIP-seq
☐	☒ Flow cytometry
☒	☐ MRI-based neuroimaging

Antibodies

Antibodies used	anti-HA (Millipore 3F10 clone, rat, 1:1000), anti-GFP (Abcam ab13970, chick, 1:2000), anti-PUM2 (Bethyl A300-202A, rabbit, 1:1000), anti-CD24-PE (BioLegend 138504, rat, 1:1000), anti-CD133-APC (BioLegend 141208, rat, 1:1000), anti-PAX6 (Covance PRB-278P, rabbit, 1:500),anti-APP (BioLegend 802803, mouse, 1:1000) and anti-LMNB1 (Santa Cruz sc-6217, goat, 1:3000).Secondary antibodies: donkey anti-rabbit IgG (LI-COR Biosciences, 926-32213, 1:10000), donkey anti-goat IgG (LI-COR Biosciences, LI-COR Biosciences 925-68074, 1:10000). Donkey anti-chicken 488 (Jackson ImmunoReseach, 703-546-155), donkey anti-mouse 488 (Thermo Scientific, A21202), donkey anti-rabbit 594 (Thermo Scientific, A21207), donkey anti-rat 647 (Thermo Scientific, A48272).
Validation	All antibodies were validated by the manufactures. The HA, GFP, PUM2, APP, LMNB1 antibodies were cross validated with Western blot,transfected fusion proteins and immuostaining. The PAX6 antibody was indirectly validated by its specific signal in the ventricular zone, and the APP antibody was indirectly validated by the similar expression pattern in previous published data. The CD24-PE and CD133-APC antibodies were indirectly validated by the specific gene expression in the RNA-Seq results.

Eukaryotic cell lines

Policy information about cell lines and Sex and Gender in Research

Cell line source(s)	HEK293FT (Fisher Scientific, Catalog number R70007)
Authentication	The cell lines we used was ordered from Fisher Scientific, and cell morphology, proliferation rate, gene expression profiles (based on our many RNA-Seq runs) are consistent with reported HEK293FT cells.
Mycoplasma contamination	The cell line has been tested negative for mycoplasma contamination.
Commonly misidentified lines (See ICLAC register)	No commonly misidentified cell lines were used

Animals and other research organisms

Policy information about studies involving animals; ARRIVE guidelines recommended for reporting animal research, and Sex and Gender in Research

Laboratory animals	CD-1 mice were purchased from Charles River, housed at the University of Chicago Animal Care Facility under 12-hour dark/12-hour light cycle and ambient temperature. The day (noon) when a vaginal plug showed was designated E0.5.
Wild animals	The study did not involve wild animals.
Reporting on sex	The sex has not been reported to affect cortical development in mouse embryos. Thus, sex of E14.5 or E15.5 embryos was not determined in this study.
Field-collected samples	No field collected samples were used in the study.
Ethics oversight	Mouse protocols, including in utero electroporation, were reviewed and approved by University of Chicago Institutional Animal Care and Use Committee (Approval #72543).

Note that full information on the approval of the study protocol must also be provided in the manuscript.

Flow Cytometry

Plots

Confirm that:

☒ The axis labels state the marker and fluorochrome used (e.g. CD4-FITC).

☒ The axis scales are clearly visible. Include numbers along axes only for bottom left plot of group (a 'group' is an analysis of identical markers).

☒ All plots are contour plots with outliers or pseudocolor plots.

☒ A numerical value for number of cells or percentage (with statistics) is provided.

Methodology

Sample preparation	HEK293FT cells were washed with 1x DPBS without Calcium or Magnesium (Gibco 14190250) 24 or 48 hours after transfection and digested with 0.25% Trypsin-EDTA (Gibco 25200114). Cells were diluted with 1x DPBS supplemented with 10% FBS and filtered through 70-µm cell strainers (Fisherbrand 578 22363548). IUE brains were dissected in cold 1xDPBS, digested with papain (Worthington Biochemical LK003150) for 15 mins in 37-degree incubator, and then resuspended in 1xDPBS with 1% BSA, filtered through 100-µm cell strainers (Fisherbrand 22363549).
Instrument	AriaIIlu BD Biosciences
Software	FACSDiva operating system
Cell population abundance	Each samples with at least 1x10^5 sorted cells for bulk RNA-seq.
Gating strategy	Cells expressing control vectors or PIE-RBPs were sorted for the top 40% of gated cells with the FITC signal or APC/PE signals into pre-chilled Trizol.

☒ Tick this box to confirm that a figure exemplifying the gating strategy is provided in the Supplementary Information.
